# The consolidation of open-source computer-assisted chemical synthesis data into a comprehensive database

**DOI:** 10.1186/s13321-025-01130-0

**Published:** 2025-12-04

**Authors:** Haris Hasic, Takashi Ishida

**Affiliations:** 1 Department of Computer Science, School of Computing, Institute of Science Tokyo, 2-12-1 Ookayama, Meguro-ku, Tokyo 152-8550 Japan; 2 Elix, Inc., 8-34 Yonbancho, Chiyoda-ku, Tokyo 102-0081 Japan

**Keywords:** Chemical synthesis, Retrosynthesis, Synthesizability, Chemical compounds, Chemical reactions, Chemical compound patterns, Chemical reaction patterns, Chemical synthesis routes

## Abstract

Over the past decade, computer-assisted chemical synthesis has resurfaced as a prominent research subject. Even though the idea of utilizing computers to assist chemical synthesis has existed for nearly as long as computers themselves, the inherent complexity repeatedly exceeded the available resources. However, recent machine learning approaches have exhibited the potential to break this tendency. The performance of such approaches is heavily dependent on data that suffers from limited quantity, quality, visibility, and accessibility, posing significant challenges to potential scientific breakthroughs. This research addresses these issues by consolidating all relevant open-source computer-assisted chemical synthesis data into a comprehensive database, providing a practical overview of the state of data in the process. The computer-assisted chemical synthesis or CaCS database is designed to be a central repository for storing and analyzing data, with the primary objective being easy integration and utilization within existing research projects. It provides the users with a programmatic interface to retrieve the data required for various tasks like predicting the outcomes of chemical synthesis and retrosynthetic analysis or retrosynthesis, estimating the synthesizability of chemical compounds, and planning and optimizing the chemical synthesis routes. The database archives the original data to ensure reusability and traceability in downstream tasks and stores the processed data in a more efficient manner. The advantages and disadvantages are highlighted through a realistic case study of how such a database would be utilized within a computer-assisted chemical synthesis research project today. The code and documentation relevant to the CaCS database are available on GitHub under the MIT license at https://github.com/neo-chem-synth-wave/ncsw-data.

**Scientific contribution:** The primary scientific contribution of this research is the consolidation of all relevant open-source computer-assisted chemical synthesis data into a comprehensive database. The database archives the original data to ensure reusability and traceability in downstream tasks, efficiently stores the processed data, and provides the users with a programmatic interface to manage and query the stored data. Rather than improving the existing or introducing new data, such a database provides a systematic overview of the existing open data sources and an easily reproducible environment for transparent processing and benchmarking purposes.

**Supplementary Information:**

The online version contains supplementary material available at 10.1186/s13321-025-01130-0.

## Introduction

The primary objective of drug and material discovery is to explore the chemical space and identify the chemical compounds of interest. A fundamental aspect of this process is chemical synthesis [1, 2]. The identified chemical compounds must be reliably and relatively efficiently synthesizable to be considered for further analysis. However, planning, executing, and optimizing viable chemical synthesis routes is challenging as it requires comprehensive chemistry knowledge and, more importantly, extensive practical experience. Relying on human expertise to this degree significantly diminishes the scalability of the traditional chemical synthesis approaches. Accordingly, computer assistance and automation are emerging as crucial drivers of progress in the present and the future [3, 4].

The idea of utilizing computers to assist chemical synthesis has existed for nearly as long as computers themselves, but the inherent complexity repeatedly exceeded the available resources [5]. This dynamic gradually changed over the past decade, sparking renewed research interest. The release of a substantial amount of open-source data in the form of the United States Patent Office (USPTO) [6–11] dataset introduced computer-assisted chemical synthesis to a broader research community. Additionally, the emergence of advanced rule-based approaches like Chematica/Synthia [12–14] demonstrated that the consistently expanding body of knowledge could be effectively and efficiently represented and utilized *in silico*. Ultimately, the successful integration of advanced machine learning and chemical synthesis concepts [15–17] opened the door for entirely data-driven approaches prevalent today. This sequence of events demonstrates that research progress is most probable when the required resources are accessible to everyone. However, as with everything, the potential is always finite. As novel approaches continue to emerge, it is becoming more evident that research progress is starting to slow down again, and the utilized data are crystallizing as the prominent limiting factor [18, 19].

**Fig. 1:**
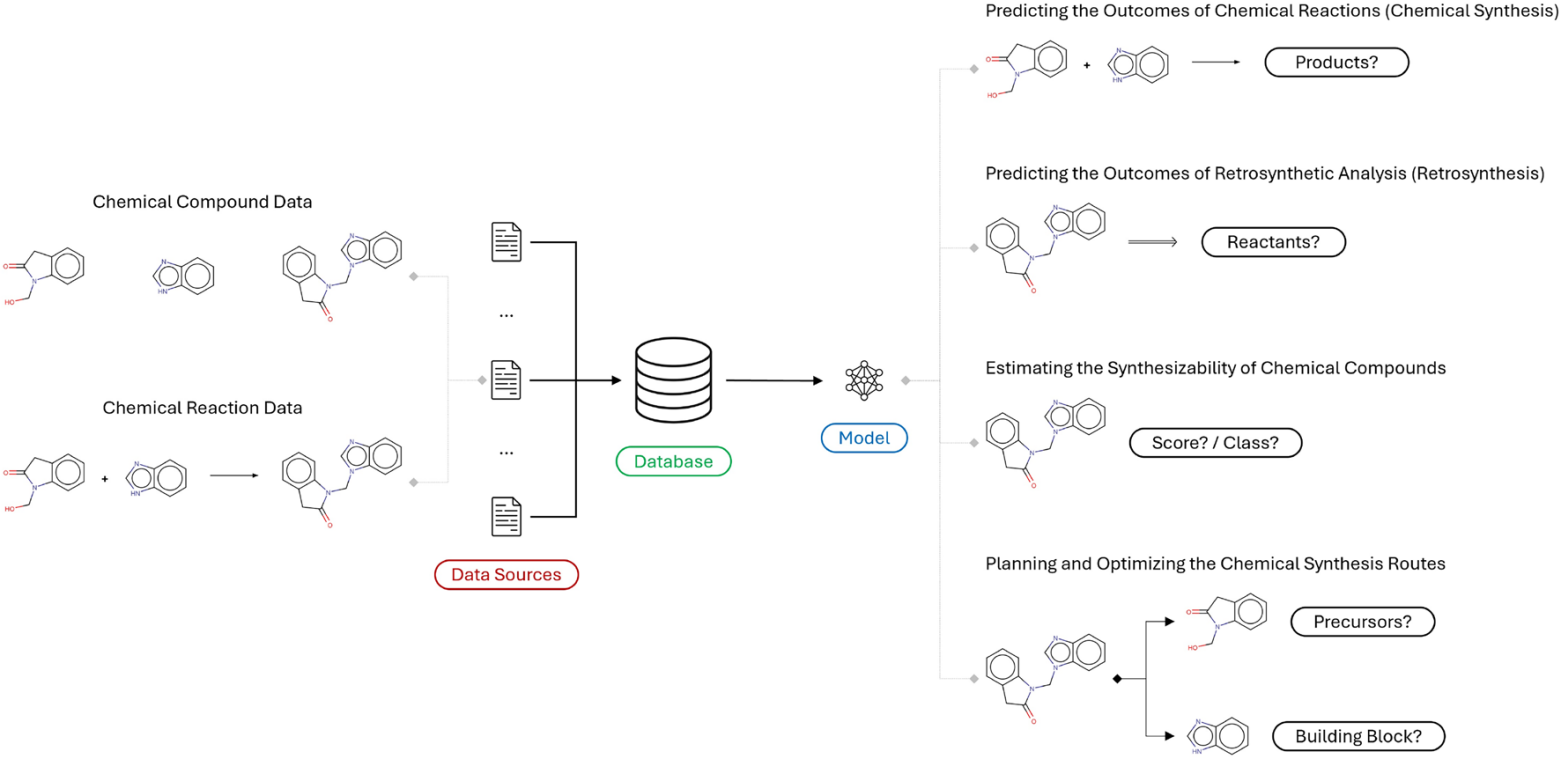
The computer-assisted chemical synthesis data and tasks. (The chemical compounds and reactions are illustrated using the Marvin [20] software version 24.3.1.)

Computer-assisted chemical synthesis encompasses various tasks, including predicting the outcomes of chemical synthesis and retrosynthetic analysis (retrosynthesis), estimating the synthesizability of chemical compounds, and planning and optimizing the chemical synthesis routes. As illustrated in Fig. 1, most of these tasks rely on two distinct types of data: chemical compound and chemical reaction data. However, these data frequently suffer from limited quantity, quality, accessibility, and visibility. While a considerable amount of quality chemical compound data is accessible through sources like ZINC [21–23] and ChEMBL [24], chemical reaction data are significantly more scarce, which is understandable considering their potential commercial value. This scarcity directly impacts the performance of computer-assisted chemical synthesis approaches, as the body of chemical reaction knowledge is known to expand and evolve rapidly over time [25]. Furthermore, the primary source of chemical reaction data (i.e., the USPTO dataset) is constructed through text mining of patent grant and application documents and is consequently relatively noisy. (e.g., invalid chemical compound structures, inaccurate chemical reaction role assignment, or incorrect or missing chemical reaction yield information) Most other relevant chemical reaction data sources are either derived from it or substantially smaller in scale and scope, offering limited additional value. These challenges are further exacerbated by the limited accessibility and visibility of open data sources, many of which are scattered across various journal publications and stored in different formats, and may not be readily available to everyone. On top of everything, considering the variety of data processing procedures, such as chemical reaction role assignment, chemical compound standardization, and chemical reaction compound atom-to-atom mapping, it is frequently unclear how to apply them consistently to ensure reproducible results. Consequently, the need for systematic curation and improved accessibility and shareability of relevant open data sources for computer-assisted chemical synthesis is more critical than ever.

The improvement and addition of open computer-assisted chemical synthesis data sources is significantly challenging, as evidenced by the Open Reaction Database (ORD) [26] and Chemical Reaction Database (CRD) [27] initiatives, which aspire to establish open-source alternatives to proprietary data sources through community-driven efforts. While such initiatives are paramount for significant progress, they provide long-term benefits as the manual input and verification of data requires expertise and time. In contrast, consolidating the existing open computer-assisted chemical synthesis data sources can provide immediate short-term benefits. For example, combining multiple smaller data sources can significantly enhance the representation of a particular chemical reaction mechanism and impact the overall performance of an approach. The consolidation can also help denoise the data and facilitate establishing and distributing universal benchmark datasets, enabling standardized and more rigorous performance comparisons. While adding and expanding the open computer-assisted chemical synthesis data sources is the utmost priority, the complementary consolidation of data sources remains critical to prolong incremental research progress in the meantime. Accordingly, this research project introduces the computer-assisted chemical synthesis (CaCS) database, which contains all relevant open-source data and infrastructure to support researchers across diverse domains working on computer-assisted chemical synthesis projects.

The CaCS database is implemented as a relational database encompassing chemical compounds, chemical compound patterns, chemical reactions, and chemical reaction patterns from various data sources to take advantage of the relational nature of these data. Namely, chemical reactions can be modeled as relationships between sets of chemical compounds. It is designed to facilitate storage space preservation and flexibility of utilization, all while retaining the original data. As a consolidated and centralized computer-assisted chemical synthesis database, the CaCS database offers insight into what and how much open-source data is realistically available today and enables single-point access for all interested parties. This research project aimed to accomplish the following key objectives: systematization of available computer-assisted chemical synthesis data sources, consolidation of data into a comprehensive database, and facilitation of access to the database. Accomplishing them through the CaCS database paves the way for more efficient and scalable solutions in the future of computer-assisted chemical synthesis research.

## Construction and content

Chemical reactions describe the transformative relationships between sets of chemical compounds, and hence, it can be argued that the only proper data type is the latter. In practice, however, a distinction must be made between chemical compounds, chemical reactions, chemical compound patterns, and chemical reaction patterns because of the different representation formats and application domains. Nevertheless, the existing relationships between these data types can be taken advantage of using a relational database. The CaCS database is doing so by storing the computer-assisted chemical synthesis data in the Simplified Molecular Input Line Entry System (SMILES) format and modeling the chemical reaction and chemical reaction pattern roles through database relationships with chemical compounds and chemical compound patterns, respectively. Assuming three distinct chemical reaction roles (i.e., chemical reaction reactant, spectator, and product compounds), this design results in a highly interconnected database. Consequently, the key aspects of the CaCS database are the supported data sources and its design and implementation.

### Data sources

**Fig. 2:**
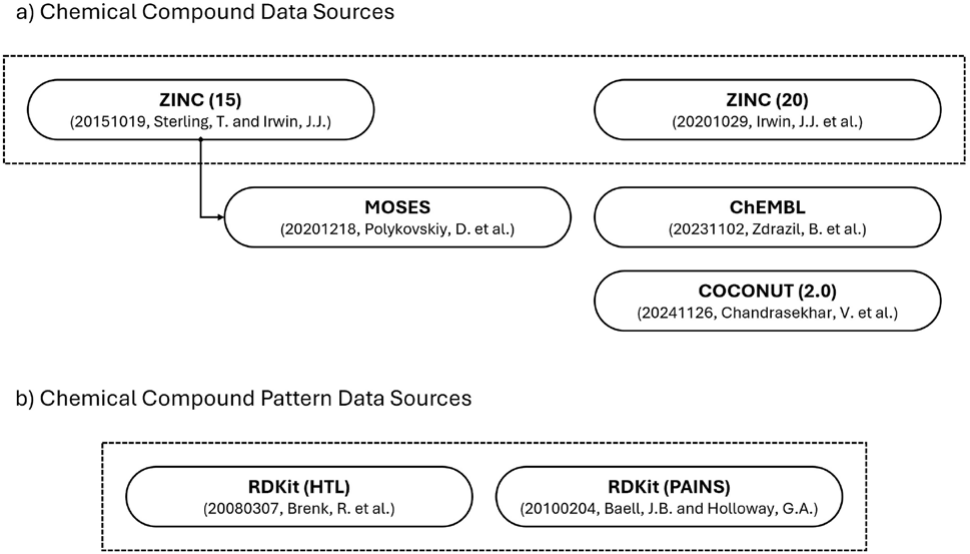
The relationships between the **a** chemical compound and **b** chemical compound pattern data sources encompassed by the CaCS database. (The arrows represent which data source is derived from which data source. The solid and dotted lines represent whether the data source is publicly available or not, respectively. The dashed lines represent a family of data sources.)

Chemical compound data are primarily utilized for building block and target chemical compound information. Building block chemical compounds are considered procurable and used as a termination condition during the planning and optimization of chemical synthesis routes. This data can be obtained directly from vendors, together with the accompanying identification and price information, but access is frequently limited to institutions with existing business or research relationships. While the ZINC [21–23] database contains a subset of building block chemical compounds, it substitutes the pricing information with a categorical availability class, limiting its utility. Target chemical compounds are used for evaluation purposes during the planning and optimization of chemical synthesis routes and are obtained from data sources with appropriate distributions. As illustrated in Fig. 2a, the CaCS database currently encompasses the ZINC, ChEMBL [24], COCONUT [28, 29], and miscellaneous [30] chemical compound data sources. Chemical compound patterns are used to filter unwanted chemical compound structures. (e.g., removing chemical compounds with functional groups that can negatively affect the chemical reaction) As illustrated in Fig. 2b, the CaCS database currently encompasses the RDKit [31, 32] chemical compound pattern data sources.

**Fig. 3:**
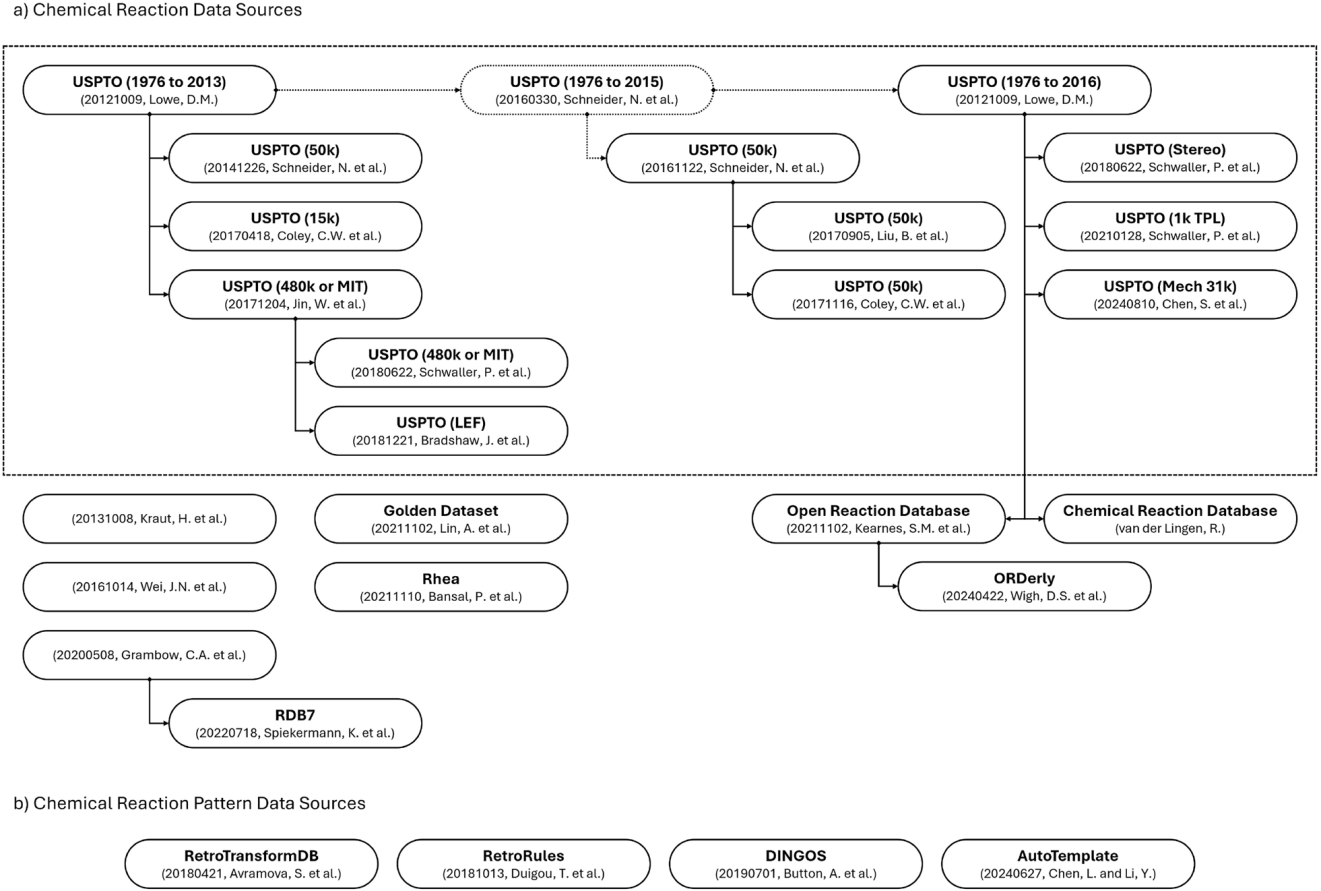
The relationships between the **a** chemical reaction and **b** chemical reaction pattern data sources encompassed by the CaCS database. (The arrows represent which data source is derived from which data source. The solid and dotted lines represent whether the data source is publicly available or not, respectively. The dashed lines represent a family of data sources.)

Chemical reaction data are primarily utilized for chemical reaction and chemical synthesis route information. Chemical reactions describe the transformative relationships between sets of chemical compounds and are used for various purposes in computer-assisted chemical synthesis. Chemical synthesis routes outline viable sequences of chemical reactions and are used for evaluation purposes during the planning and optimization of chemical synthesis routes. As illustrated in Fig. 3a, the CaCS database currently encompasses the USPTO [6, 7, 9–11, 33–42], ORD [26], CRD [27], Rhea [43], and miscellaneous [44–49] chemical reaction data sources. Chemical reaction pattern data are primarily utilized for chemical reaction transformation information. Chemical reaction transformations outline a transformative relationship between two sets of chemical compound patterns, which can be represented in the forward and backward directions. The chemical reaction patterns are matched against building block or target chemical compounds to establish whether a chemical reaction is theoretically possible. As illustrated in Fig. 3b, the CaCS database currently encompasses the RetroRules [50] and miscellaneous [51–53] chemical reaction pattern data sources.

The downloading, extraction, and formatting of open-source computer-assisted chemical synthesis data is also available as a standalone package called data_source on GitHub under the MIT license at https://github.com/neo-chem-synth-wave/data-source.

### Database

**Fig. 4:**
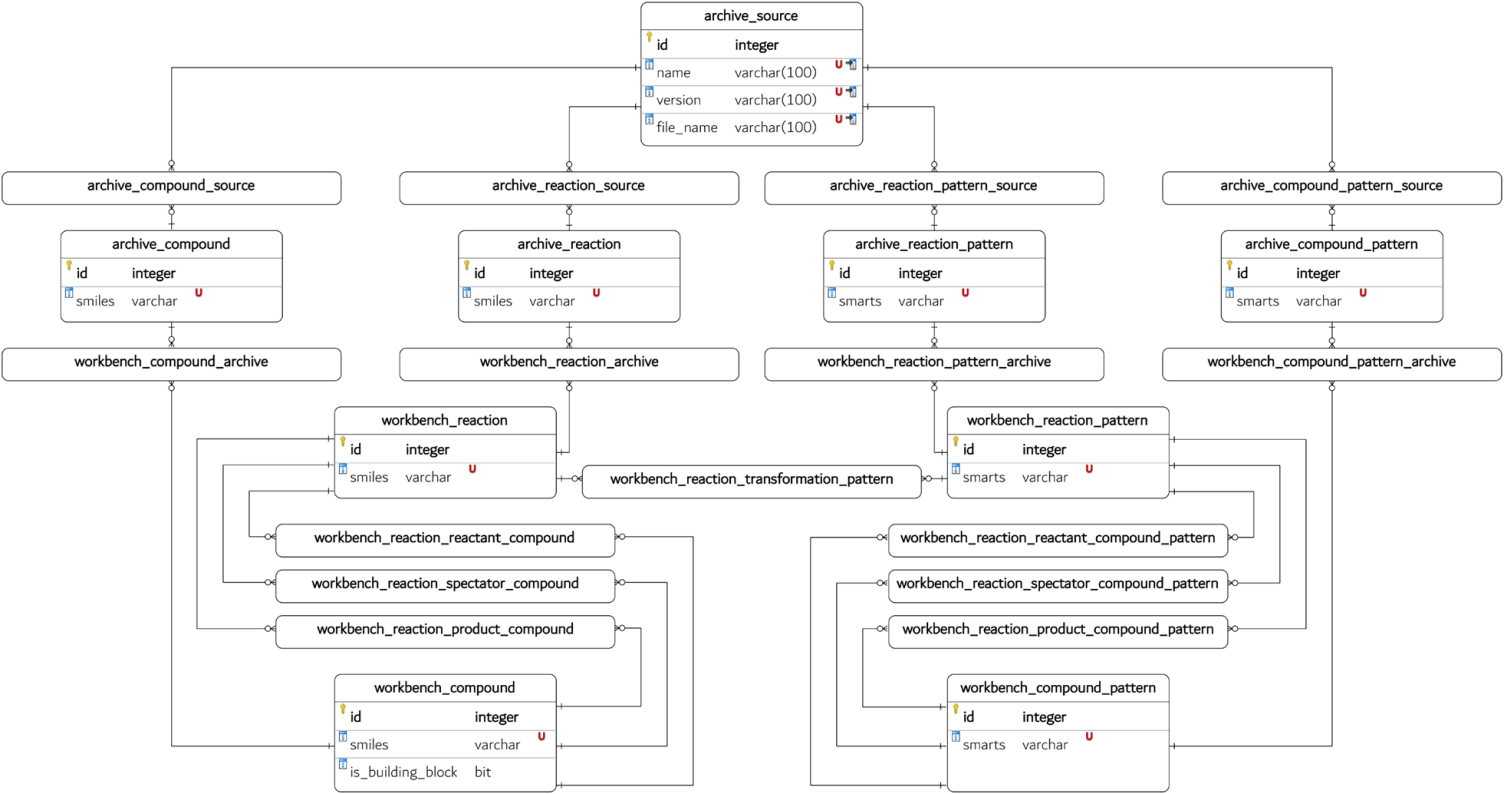
The ERD of the CaCS database. (The ERD is illustrated using the Visual Paradigm [54] software version 17.2.)

The CaCS database is designed and implemented as a relational database to take advantage of the relational nature of computer-assisted chemical synthesis data. As outlined in the Entity Relationship Diagram (ERD) illustrated in Fig. 4, the CaCS database consists of 24 tables in total. The majority of the data is stored in the following 9 database tables: *archive_source*, *archive_compound*, *archive_reaction*, *archive_compound_pattern*, *archive_reaction_pattern*, *workbench_compound*, *workbench_reaction*, *workbench_compound_pattern*, and *workbench_reaction_pattern*. The remaining 16 database tables are association tables where the many-to-many relationship information is stored. Currently, that information is limited to the foreign keys of the relevant database tables, but it may be expanded in the future. (e.g., adding chemical reaction spectator compound role details) This design choice facilitates storage space preservation and flexible use of the data, with each table serving a distinct purpose within the overall structure of the database.

The CaCS database is organized into two distinct namespaces: archive and workbench. The rationale behind this organization is to preserve the original data extracted from various sources, enabling downstream versions to be traced back to the origin. This mechanism can improve the reproducibility and explainability of downstream approaches and facilitate the preservation of storage space by allowing duplicates and invalid entries in the workbench to be filtered out. Furthermore, only the data of interest may be migrated from the archive to the workbench in projects where the entirety of the data is not required. Selecting only the relevant data in this manner can positively affect the performance of the database and the downstream approaches. Consequently, the archive database tables should be ideally used only for inserting, while the workbench database tables should be generated anew for every research project.

The archive database tables are centered around the *archive_source* table, which stores the information about the source from which the data is retrieved. It is connected to the *archive_compound*, *archive_reaction*, *archive_compound_pattern*, and *archive_reaction_pattern* tables using many-to-many relationships because the data can have multiple sources and *vice versa*. Similarly, these tables are connected to the *workbench_compound*, *workbench_reaction*, *workbench_compound_pattern*, and *workbench_reaction_pattern* tables, which store the standardized version of the data. The *workbench_reaction* table is connected to the *workbench_reaction_pattern* table to establish a many-to-many relationship detailing which chemical reaction pattern applies to which chemical reaction. The *workbench_compound* and *workbench_reaction* tables are connected using three association tables to denote the established chemical reaction roles. The same design choice is made for the *workbench_compound_pattern* and *workbench_reaction_pattern* database tables.

The initial version of the CaCS database utilizes the SQLite [55] engine for ease of use and agile prototyping purposes. The programmatic interface to the database is implemented in Python [56] and SQLAlchemy [57], utilizing the object-relational mapping (ORM) technique. All interactions with the database are managed utilizing Python code, which allows for advanced security and quality assurance measures. The create, read, update, and delete (CRUD) operations are implemented with transactional integrity guidelines in mind. While the data in the archive tables of the database is stored as is, the standardization and migration of the data to the workbench tables of the database are specified by the user. The interface does not provide built-in standardization procedures, which may vary significantly depending on the research project. However, there are example scripts that illustrate how it can be done utilizing state-of-the-art libraries like RDKit [58], RXNMapper [40], and RDChiral [59]. This design choice allows for easily reproducible environments for data processing and benchmarking.

The downloading, extraction, and formatting of open-source computer-assisted chemical synthesis data, as well as the construction, management, and querying of the CaCS database, are available as a package called ncsw_data on GitHub under the MIT license at https://github.com/neo-chem-synth-wave/ncsw-data.

## Utility and discussion

The CaCS database is designed and implemented to alleviate and standardize the data collection and processing in computer-assisted chemical synthesis tasks. Given the variety of data processing procedures, such as chemical reaction role assignment, chemical compound standardization, and chemical reaction compound atom-to-atom mapping, it is frequently unclear how to apply them consistently to maximize comparability. Consequently, approaches for the same tasks are rendered incomparable because the data is processed differently. Using a centralized database that allows the users to define the processing pipelines themselves while being easily shareable allows for more transparency in benchmarking. The database can also be utilized to access all available open-source data and test the limits of the developed approaches. Such a scenario is investigated through a case study illustrating the current state of computer-assisted chemical synthesis data.

### User interface

The only currently available user interface to the CaCS database is programmatic, meaning that the users interact through code rather than a command line or graphical user interface. While advanced and more user-friendly interfaces are part of future development, the primary objective of the database is easy integration and utilization within existing research projects, which is best achieved programmatically. The current user interface supports the downloading, extraction, and formatting of data, as well as the construction, management, and querying of the database. Accordingly, the users can easily retrieve and directly utilize computer-assisted chemical synthesis data from various open sources. If more advanced handling and analysis of the data is required, the users can establish and utilize a version of the database that best fits the requirements. Irrespective of the utilization details, the CaCS database is convenient for any computer-assisted chemical synthesis project.

The user interface supports three alternatives for the downloading, extraction, and formatting of a specific version of computer-assisted chemical synthesis data from a specific source. The first alternative is by importing and utilizing the individual data source utility classes:
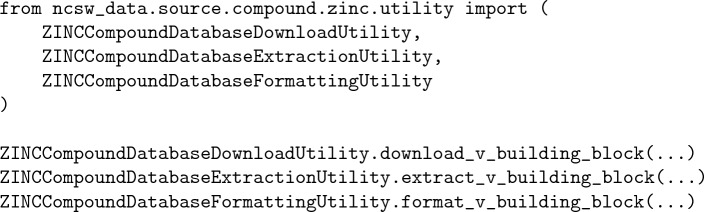


It is best suited when users are interested in a single data source and prefer a less structured but more efficient interaction. The second alternative is by importing and utilizing the individual data source classes:
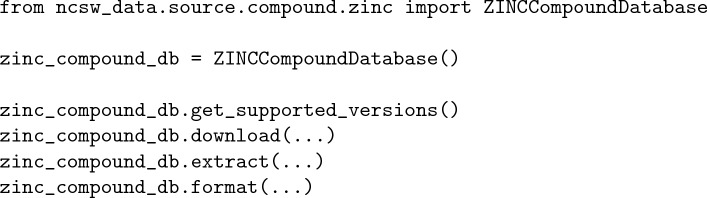


Similar to the previous alternative, it is best suited when users are interested in a single data source and prefer a more structured interaction. The third alternative is by importing and utilizing the data source category classes:
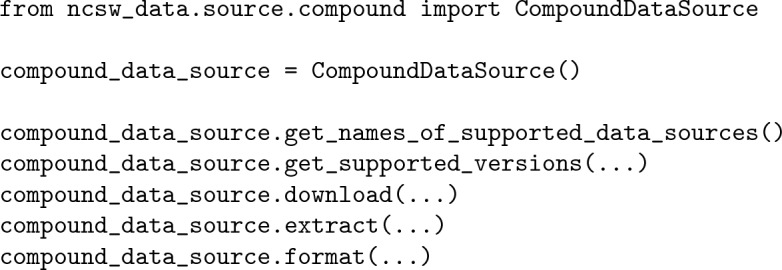


This alternative is best suited when users are interested in multiple data sources from the same category and prefer a more structured interaction. Ultimately, each alternative has advantages and disadvantages, and users can decide which alternative is preferred.

The CaCS database is constructed and managed in three stages: inserting data into the archive tables, migrating data from the archive to the workbench tables, and extracting and storing the workbench chemical reaction transformation patterns. The first two stages are performed for all data types, while the last stage is performed only for chemical reactions and chemical reaction patterns:
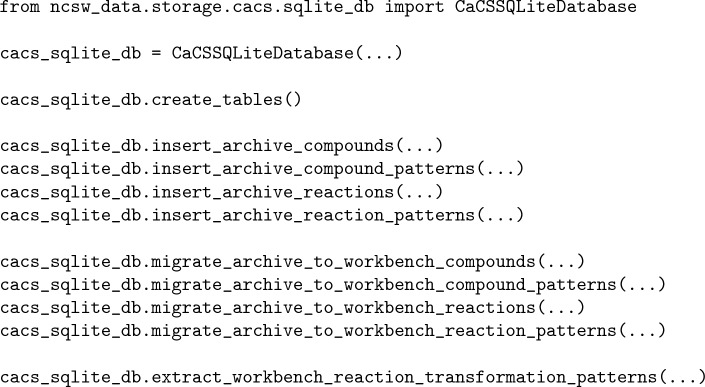


First, the database tables are created if necessary. Next, the original data are inserted into the archive tables. Finally, the data are migrated to the workbench tables. (i.e., standardized and processed utilizing user-specified procedures addressing specific requirements and stored in the appropriate workbench tables) Optionally, the chemical reaction pattern data are extracted from the chemical reaction data in the workbench utilizing user-specified procedures and stored appropriately. After the database is constructed, the tables can be queried utilizing predefined class methods as well as a general query class method for the execution of more complex queries:
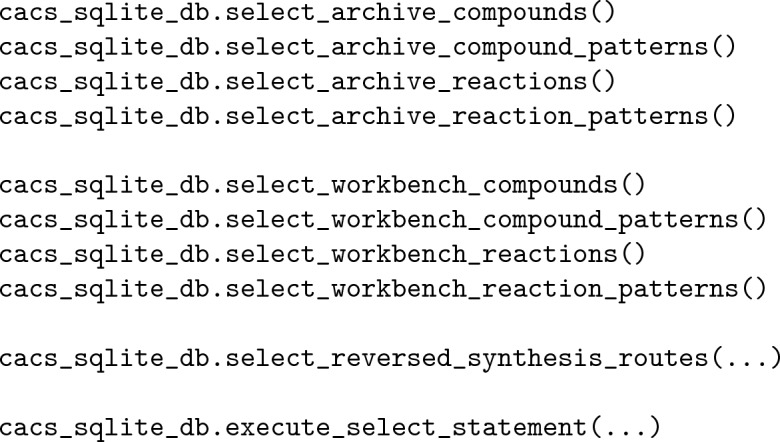


Furthermore, the chemical synthesis routes derived from the chemical reaction data can be extracted utilizing a predefined common table expression (CTE) query. While the CaCS database does not introduce new computer-assisted chemical synthesis data, consolidating and structuring the existing data and providing such an interface significantly increases its utility.

### Discussion

The CaCS database is designed to be a central repository for storing and utilizing computer-assisted chemical synthesis data. Its primary objective is to consolidate data from various open sources and allow users to access it in the original and processed forms. In this way, the users can utilize the database to obtain a specific subset of the data (e.g., only specific chemical reaction mechanisms) or an already established dataset (e.g., USPTO-50k [35]) relevant to their research project. Moreover, the processed data can be easily related to the source for practical purposes. (e.g., retrieving the document in which a specific chemical reaction mechanism is introduced) The CaCS database is also designed to simplify frequent computer-assisted chemical synthesis tasks, like interacting with the individual chemical reaction compounds or extracting chemical synthesis routes. Ultimately, the fact that it is a relational database makes the set of interactions with the data considerably more comprehensive than simply using flat files.

Having the flexibility that such a database provides is significant, especially for novice researchers. The easier it is to utilize and understand, the easier it is for users to get familiar with the state of data in this research subject. Users can only download the original data and skip using the CaCS database altogether if they are only interested in individual data sources. However, if the project requires more advanced data handling and analysis, they can use the database and easily share their processed data with other researchers. For example, utilizing the mapped chemical reaction SMILES strings and the canonicalized versions of the reactants, spectators, and product chemical compounds (e.g., inputting the data into a machine learning model) becomes as simple as a single for loop:
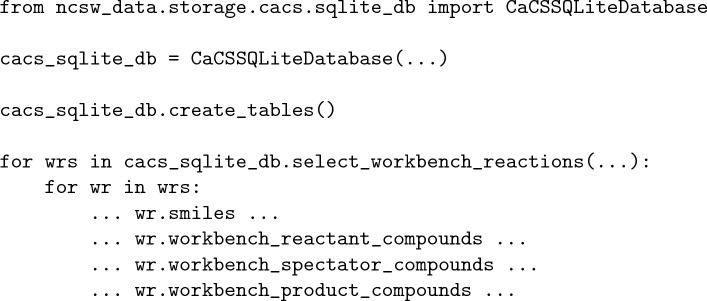


In contrast, when working directly with chemical reaction SMILES strings, the parsing, conversion, and standardization of the individual chemical compounds must be performed every time. Advanced users also benefit from the same simple calls to the database or the ability to perform more advanced analysis, such as analyzing chemical synthesis routes. Ultimately, the CaCS database has advantages and disadvantages, but considering that no similar solutions exist as of the time of writing, its actual practicality remains to be determined. While similar initiatives like ORD [26] and CRD [27] exist, they are primarily focused on improving the existing or adding new data while doing little to optimize large-scale access for downstream tasks. Usually, the entirety of the data is available for download through dump files or functions that can frequently be inefficient. In that sense, the CaCS database is the first solution that consolidates the data as well as alleviates delivery to the users.

The advantages and disadvantages of the CaCS database can be briefly summarized through simplicity and specificity. The database is implemented utilizing the SQLite [55] engine, making it easy to set up and integrate within existing research projects. Additionally, the types that are stored in the database are limited to numerical and textual information, which lessens the required storage space. On the other hand, the performance of the database using such a simple engine is not as good as it could be using a more advanced engine like PostgreSQL [60], and it does not benefit from extension cartridges [58, 61] tailored explicitly for databases of this type. That is especially noticeable when substantial data is contained, and the queries get more complicated. (e.g., the CTE queries for chemical synthesis routes) Also, the database lacks specificity and does not support useful tools like similarity searching.

### Case study

As illustrated in Fig. 1, computer-assisted chemical synthesis encompasses various tasks, including predicting the outcomes of chemical synthesis and retrosynthesis, estimating the synthesizability of chemical compounds, and planning and optimizing the chemical synthesis routes. Most of these tasks are entirely data-driven, and their performance significantly depends on the quantity and quality of the available data. This case study aims to provide a clear overview of how much open-source data is realistically available for computer-assisted chemical synthesis research projects. This overview is realized by retrieving, storing, and analyzing the relevant data utilizing the CaCS database as a central repository. The database consists of the primary data sources to maximize the amount of relevant data historically and follows the most frequently utilized strategies for data processing. Ultimately, the overview is illustrated through an in-depth analysis of the available data, and the additional details are outlined in the Case study implementation details section of the Supplementary information.

According to Table 1, the total number of archive chemical reactions is 3,294,548, of which 3,287,740 are associated with at least one workbench chemical reaction. A utilization rate of 99.79% might imply a high quality of data, but considering that the total number of workbench chemical reactions is 2,448,637, this is not the case. The calculated chemical reaction expansion factor (WR/UAR) of 0.743 implies a high level of redundancy in the data. The primary contributor of workbench chemical reaction data is the USPTO [6, 10] dataset with 1,437,750 (58.72%), followed by the CRD [27] with 747,264 (30.52%), the ORD [26] with 260,924 (10.66%), and miscellaneous [44, 45, 47] data sources with 2,699 (0.1%) workbench chemical reactions. While the ORD and CRD both contain data from the USPTO dataset, it is evident that the primary objective of the ORD is to improve data quality, while the primary objective of the CRD is to improve data quantity. The additional statistics on the chemical compounds and chemical reaction patterns are outlined in Table S1 and Table S2 of the Case study analysis results section of the Supplementary information.

The number of reactant compounds in the workbench chemical reactions varies from 1 to 12, with most chemical reactions having 2 (69.88%), 1 (21.66%), or 3 (6.31%) reactant compounds, respectively. If these data are integrated with the available building block information, 876,554 or 35.8% of workbench chemical reactions do not include building block reactant compounds, while 469,697 or 19.18% only include building block reactant compounds. The remaining 1,102,386 or 45.02% of workbench chemical reactions contain some building block reactant compounds. A total of 236,816 (9.67%) of workbench chemical reactions contain a building block product compound, and 739,516 (30.2%) of workbench chemical reactions contain product compounds that are also involved as reactant compounds in other workbench chemical reactions. This distribution offers insight into how valuable the chemical reaction data can be for predicting the outcomes of chemical synthesis and retrosynthesis, as well as planning and optimizing the chemical synthesis routes. On the one hand, the workbench chemical reactions with building block reactant chemical compounds imply increased opportunities to explore the chemical space in the forward direction and shorter chemical synthesis routes. On the other hand, many workbench chemical reactions with building block product compounds imply less valuable chemical transformation patterns in the backward direction. The additional statistics on the workbench chemical reaction reactant, product, and building block compounds are illustrated in Figure S1 and Figure S2 of the Case study analysis results section of the Supplementary information.

**Table 1:** The total number of archive and workbench chemical reactions and the exclusive number of archive and workbench chemical reactions per data source in the CaCS database

Data source	AR^a^	UAR^b^	UAR^b^ (%)	WR^c^	EF^d^
	3,294,548	3,287,740	99.79	2,448,637	0.743
USPTO [6, 10]	1,484,441	1,484,069	99.98	1,437,750	0.969
ORD [26]	507,136	501,464	98.88	260,924	0.520
CRD [27]	1,300,871	1,300,112	99.94	747,264	0.575
Miscellaneous [44, 45, 47]	2,100	2,095	99.76	2,699	1.288

^a^Archive Chemical Reactions;

^b^Utilized Archive Chemical Reactions (Related to WR in the database.)

^c^Workbench Chemical Reactions

^d^Expansion Factor (WR/UAR)

**Fig. 5:**
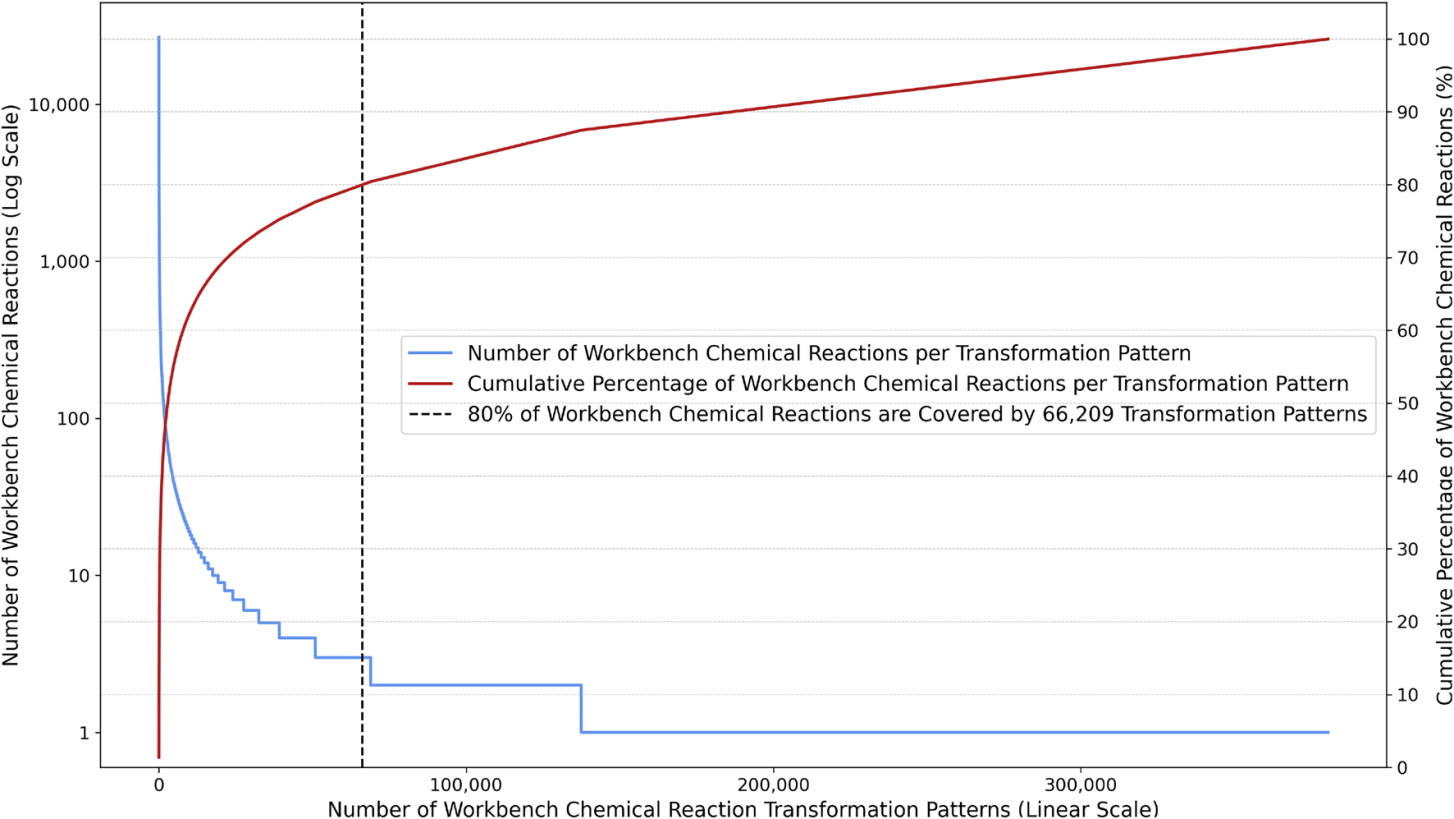
The number and cumulative percentage of workbench chemical reactions per chemical reaction transformation pattern in the CaCS database

The total number of workbench chemical reaction patterns is 383,296, of which 380,414 (99.25%) are extracted from the workbench chemical reactions, while the rest are migrated from the archive chemical reaction patterns. These chemical reaction patterns are successfully extracted from 1,940,853 (79.26%) of workbench chemical reactions, and the extraction failed for 507,784 (20.74%) of workbench chemical reactions, primarily because of chemical reaction reactive sites consisting of multiple disconnected components. As illustrated in Fig. 5, 80% of workbench chemical reactions are covered by 66,209 (17.4%) chemical reaction patterns. Most workbench chemical reactions have unique chemical reaction patterns, representing a significant challenge for computer-assisted chemical synthesis approaches. For example, the quality of retrosynthesis models heavily depends on striking a balance between what chemical reaction patterns are included. In the case of template-based (e.g., 3N-MCTS [17]) retrosynthesis, the models suffer from either an incomplete knowledge base or a lack of training data if chemical reaction patterns with a single occurrence are excluded or not, respectively. In the case of semi-template-based (e.g., GraphRetro [62]) and template-free (e.g., NLP Transformer [63]) retrosynthesis, the models suffer from a general lack of training data. Additional statistics about workbench chemical reaction transformation patterns are illustrated in Figure S3 of the Case study analysis results section of the Supplementary information.

As of the time of writing, there are no available open-source datasets containing chemical synthesis route information that can be traced back to the source. Accordingly, the CaCS database allows for deriving chemical synthesis route information from chemical reaction data by recursively searching the database for instances where the target chemical compound is involved in a chemical reaction as a product compound. While these are not chemical synthesis routes that can be traced back to specific literature, they are theoretically possible and hold a similar weight as the extracted chemical reaction transformation patterns. The only downside is that, especially when working with data at this scale, the CTE queries are not well suited for execution and are relatively slow, considering their recursive nature. This lack of performance makes the construction of a global chemical synthesis route dataset impractical, at least utilizing the SQLite [55] engine. Nevertheless, the possibility of extracting chemical synthesis routes for particular target chemical compounds is supported.

### Future development

The short-term future development of the CaCS database will focus on two main activities: maintaining and adding data sources and improving the database design and interface. The activities centered around the data sources will include maintaining and improving the handling of the existing data sources (e.g., updating download links and improving the parsing of files) and adding new data sources. The activities centered around the database will include improving the amount and type of information stored in the database (e.g., adding yield information), improving the performance of existing CRUD methods, and adding new CRUD methods. The long-term future development will focus on designing and constructing the CaCS database utilizing the PostgreSQL [60] engine together with appropriate extension cartridges [58, 61] for chemical information databases. The projected outcome of these future improvement activities is two different versions of the CaCS database, where the current version utilizing the SQLite [55] engine is designed for ease of use and integration, (e.g., in open-source research projects) and the version utilizing the PostgreSQL engine is designed for performance and advanced analysis. (e.g., in corporate research and development projects) Considering this is an open-source research project with limited time and resources, this projected outcome is likely to take some time to materialize.

## Conclusion

Computer-assisted chemical synthesis has repeatedly resurfaced as a prominent research subject in cheminformatics, but the inherent complexity consistently exceeded the available resources. Today, the continued convergence of intellectual and financial incentives and novel technology concepts suggests that significant breakthroughs are imminent. However, for any breakthrough to materialize, the necessary prerequisites must be met, with appropriate data being the most critical in this case. Accordingly, this research introduces the CaCS database, a centralized repository for open-source computer-assisted chemical synthesis data. The primary objective of this database is the effective and efficient consolidation and utilization of data within existing research projects. Unlike initiatives such as the ORD [26] and CRD [27], it is the first database to exclusively focus on consolidating and distributing rather than improving or adding the data.

As with everything else, the CaCS database has advantages and disadvantages, both of which can be briefly summarized through simplicity and specificity. The database is implemented utilizing the SQLite [55] engine, making it general and easy to set up and integrate within existing research projects. On the other hand, the performance is not as good as it could be using a more advanced engine like PostgreSQL [60], and it does not benefit from extension cartridges [58, 61] tailored explicitly for databases of this type. The immediate future development of the database will be centered around maintaining and adding data sources, as well as improving the design and programmatic interface of the database. The development of the PostgreSQL version of the database will also commence in the meantime. Ultimately, the CaCS database can be convenient in any computer-assisted chemical synthesis research project.

## Supplementary Information

Supplementary material 1.

## Data Availability

The data_source package and relevant data, scripts, and documentation are available on GitHub under the MIT license at https://github.com/neo-chem-synth-wave/data-source. The ncsw_data package and relevant scripts, notebooks, and documentation are available on GitHub under the MIT license at https://github.com/neo-chem-synth-wave/ncsw-data. The database version utilized in the case study, as well as the database version encompassing the archive data from all sources supported by the data_source and ncsw_data packages as of the time of writing, are available on Figshare under the MIT license at https://figshare.com/articles/dataset/The_Computer-assisted_Chemical_Synthesis_CaCS_Database/29124725.

## Abbreviations

USPTO: United States Patent Office
ORD: Open reaction database
CRD: Chemical reaction database
CaCS: Computer-assisted chemical synthesis
SMILES: Simplified molecular input line entry system
ERD: Entity relationship diagram
ORM: Object-relational mapping
CRUD: Create, read, update, and delete
CTE: Common table expression
