# Supplementary material for: The consolidation of open-source computer-assisted chemical synthesis data into a comprehensive database
Source: J Cheminform. 2025 Dec 4;18:4. doi: 10.1186/s13321-025-01130-0 (PMC12781325; doi:10.1186/s13321-025-01130-0)
Supplement: Supplementary file 1 — Supplementary material 1. [file 13321_2025_1130_MOESM1_ESM.pdf]

Supplementary information for: The consolidation of open-source computer-assisted chemical synthesis data into a comprehensive database

Haris Hasic[1,2*] and Takashi Ishida[1]

[1*]Department of Computer Science, School of Computing, Institute of Science Tokyo, 2-12-1 Ookayama, Meguro-ku, Tokyo 152-8550, Japan.
[2]Elix, Inc., 8-34 Yonbancho, Chiyoda-ku, Tokyo 102-0081, Japan.

*Corresponding author(s). E-mail(s): hasic@cb.cs.titech.ac.jp;

S1 Case study implementation details

The CaCS database is implemented in two stages: downloading, extraction, and formatting of the data, and the construction and management of the database. First, the original computer-assisted chemical synthesis data files are downloaded directly from the source. Next, any archive files are extracted as necessary. Finally, the contents of the relevant data files are aggregated and stored in a single file, which concludes the first stage. In the second stage, the data is first imported into the archive tables of the database. Next, the data is migrated from the archive to the workbench tables of the database, utilizing a data processing function specified by the user. Finally, the chemical reaction patterns are extracted from the workbench chemical reactions and stored in the appropriate tables of the database, which concludes the second stage. After both stages are concluded, the database can be utilized in a plug-and-play manner by importing the resulting database file.

The chemical compound data are selected from the ZINC [1] database to encompass building block information. No chemical compound pattern data is included because it is irrelevant to the case study. The chemical reaction data are selected from the USPTO [2, 3], ORD [4], CRD [5], and miscellaneous [6–8] data sources. The chemical reaction pattern data are selected from miscellaneous [9–11] data sources for completeness. The chemical compounds are sanitized and canonicalized before the migration to the workbench. The chemical reactions are first parsed into reactant, spectator, and product chemical compounds by splitting the SMILES string using the symbol $>$ as

the delimiter. Next, the chemical compounds are sanitized and canonicalized, and the reactant and spectator compounds are combined before atom-to-atom mapping using the RXNMapper [12] library, eliminating spectator compounds altogether. Ultimately, the mapped chemical reactions are split into single-product chemical reactions where only the relevant reactant chemical compounds (*i.e.*, reactant chemical compounds with at least one atom map number in common with the product chemical compound) are kept. The chemical reaction templates are extracted utilizing the RDChiral [13] library and checked for applicability before migrating to the workbench. The chemical compound patterns and chemical reaction patterns are processed minimally.

S2 Case study analysis results

Table S1 The total number of archive and workbench chemical compounds and the exclusive number of archive and workbench chemical compounds per data source in the CaCS database.

**Data Source**	**AC**[1]	**UAC**[2]	**UAC**[2] **(%)**	**WC**[3]
	1,953,848	1,953,841	99.99	1,953,841
ZINC (BB 50) [1]	1,334,114	1,334,107	99.99	1,334,107
ZINC (BB 40) [1]	616,442	616,442	100.0	616,442
ZINC (BB 30) [1]	3,292	3,292	100.0	3,292

[1] Archive Chemical Compounds

[2] Utilized Archive Chemical Compounds (Related to WC in the database.)

[3] Workbench Chemical Compounds

Table S2 The total number of archive and workbench chemical reaction patterns and the exclusive number of archive and workbench chemical reaction patterns per data source in the CaCS database.

**Data Source**	**ARP**[1]	**UARP**[2]	**UARP**[2] **(%)**	**WRP**[3]
	2,882	2,882	100.0	2,882
Miscellaneous (RetroTransformDB) [9]	105	105	100.0	105
Miscellaneous (DINGOS) [10]	64	64	100.0	64
Miscellaneous (AutoTemplate) [11]	2,713	2,713	100.0	2,713

[1] Archive Chemical Reaction Patterns

[2] Utilized Archive Chemical Reaction Patterns (Related to WRP in the database.)

[3] Workbench Chemical Reaction Patterns

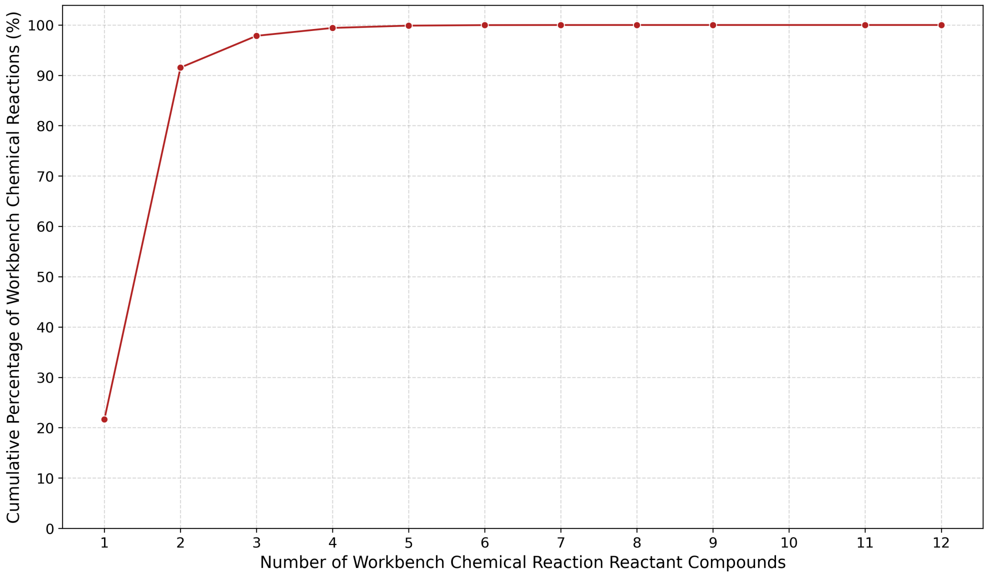

Fig. S1 The cumulative percentage of workbench chemical reactions per number of reactant compounds in the CaCS database.

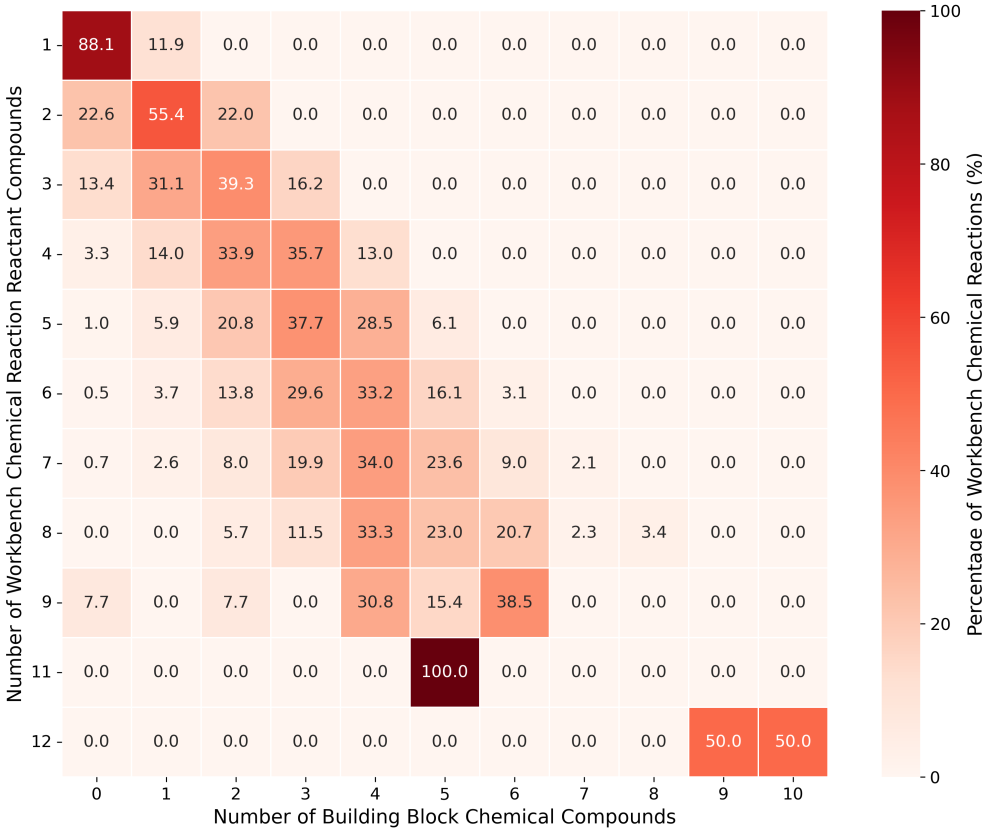

Fig. S2 The percentage of workbench chemical reactions per number of reactant and building block compounds in the CaCS database.

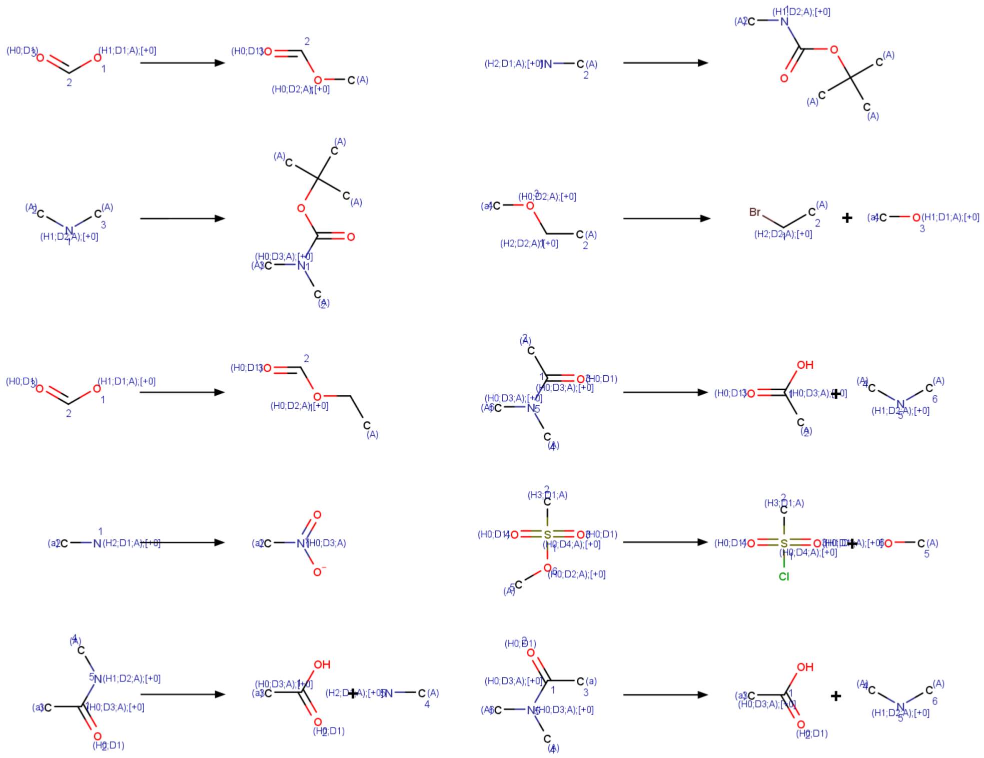

Fig. S3 The top ten most common workbench chemical reaction patterns in the CaCS database.

References

[1] Irwin, J.J., Tang, K.G., Young, J., Dandarchuluun, C., Wong, B.R., Khurelbaatar, M., Moroz, Y.S., Mayfield, J., Sayle, R.A.: Zinc20 - a free ultralarge-scale chemical database for ligand discovery. Journal of Chemical Information and Modeling **60**(12), 6065–6073 (2020) https://doi.org/10.1021/acs.jcim.0c00675

[2] Lowe, D.M.: Extraction of chemical structures and reactions from the literature. Phd thesis, Apollo - University of Cambridge Repository (2012). https://doi.org/10.17863/CAM.16293

[3] Lowe, D.M.: Chemical reactions from US patents (1976-Sep2016) (2017). https://doi.org/10.6084/m9.figshare.5104873.v1

[4] Kearnes, S.M., Maser, M.R., Wleklinski, M., Kast, A., Doyle, A.G., Dreher, S.D., Hawkins, J.M., Jensen, K.F., Coley, C.W.: The open reaction database. Journal of the American Chemical Society **143**(45), 18820–18826 (2021) https://doi.org/10.1021/jacs.1c09820

[5] van der Lingen, R.: Home to the Chemical Reaction Database. https://kmt.vander-lingen.nl. Accessed on May 25th, 2025

[6] Kraut, H., Eiblmaier, J., Grethe, G., Löw, P., Matuszczyk, H., Saller, H.: Algorithm for reaction classification. Journal of Chemical Information and Modeling **53**(11), 2884–2895 (2013) https://doi.org/10.1021/ci400442f

[7] Wei, J.N., Duvenaud, D., Aspuru-Guzik, A.: Neural networks for the prediction of organic chemistry reactions. ACS Central Science **2**(10), 725–732 (2016) https://doi.org/10.1021/acscentsci.6b00219

[8] Lin, A., Dyubankova, N., Madzhidov, T.I., Nugmanov, R.I., Verhoeven, J., Gimadiev, T.R., Afonina, V.A., Ibragimova, Z., Rakhimbekova, A., Sidorov, P., Gedich, A., Suleymanov, R., Mukhametgaleev, R., Wegner, J., Ceulemans, H., Varnek, A.: Atom-to-atom mapping: A benchmarking study of popular mapping algorithms and consensus strategies. Molecular Informatics **41**(4), 2100138 (2022) https://doi.org/10.1002/minf.202100138

[9] Avramova, S., Kochev, N., Angelov, P.: Retrotransformdb: A dataset of generic transforms for retrosynthetic analysis. Data **3**(2) (2018) https://doi.org/10.3390/data3020014

[10] Button, A., Merk, D., Hiss, J.A., Schneider, G.: Automated de novo molecular design by hybrid machine intelligence and rule-driven chemical synthesis. Nature Machine Intelligence **1**(7), 307–315 (2019) https://doi.org/10.1038/s42256-019-0067-7

[11] Chen, L., Li, Y.: Autotemplate: Enhancing chemical reaction datasets for machine

learning applications in organic chemistry. Journal of Cheminformatics **16**(1), 74 (2024) https://doi.org/10.1186/s13321-024-00869-2

[12] Schwaller, P., Hoover, B., Reymond, J., Strobelt, H., Laino, T.: Extraction of organic chemistry grammar from unsupervised learning of chemical reactions. Science Advances **7**(15), 4166 (2021) https://doi.org/10.1126/sciadv.abe4166

[13] Coley, C.W., Green, W.H., Jensen, K.F.: Rdchiral: An rdkit wrapper for handling stereochemistry in retrosynthetic template extraction and application. Journal of Chemical Information and Modeling **59**(6), 2529–2537 (2019) https://doi.org/10.1021/acs.jcim.9b00286
